# A Quantitative Proteomic Profile of the Nrf2-Mediated Antioxidant Response of Macrophages to Oxidized LDL Determined by Multiplexed Selected Reaction Monitoring

**DOI:** 10.1371/journal.pone.0050016

**Published:** 2012-11-16

**Authors:** Caroline S. Kinter, Jillian M. Lundie, Halee Patel, Paul M. Rindler, Luke I. Szweda, Michael Kinter

**Affiliations:** 1 Free Radical Biology and Aging Research Program, Oklahoma Medical Research Foundation, Oklahoma City, Oklahoma, United States of America; 2 Department of Biochemistry and Molecular Biology, University of Oklahoma Medical Center, Oklahoma City, Oklahoma, United States of America; 3 Donald W. Reynolds Aging Research Program, University of Oklahoma Medical Center, Oklahoma City, Oklahoma, United States of America; Maastricht University, The Netherlands

## Abstract

The loading of macrophages with oxidized low density lipoprotein (LDL) is a key part of the initiation and progression of atherosclerosis. Oxidized LDL contains a wide ranging set of toxic species, yet the molecular events that allow macrophages to withstand loading with these toxic species are not completely characterized. The transcription factor nuclear factor (erythroid-derived 2)-like 2 (Nrf2) is a master regulator of the cellular stress response. However, the specific parts of the Nrf2-dependent stress response are diverse, with both tissue- and treatment-dependent components. The goal of these experiments was to develop and use a quantitative proteomic approach to characterize the Nrf2-dependent response in macrophages to oxidized LDL. Cultured mouse macrophages, the J774 macrophage-like cell line, were treated with a combination of oxidized LDL, the Nrf2-stabilizing reagent tert- butylhydroquinone (tBHQ), and/or Nrf2 siRNA. Protein expression was determined using a quantitative proteomics assay based on selected reaction monitoring. The assay was multiplexed to monitor a set of 28 antioxidant and stress response proteins, 6 housekeeping proteins, and 1 non-endogenous standard protein. The results have two components. The first component is the validation of the multiplexed, quantitative proteomics assay. The assay is shown to be fundamentally quantitative, precise, and accurate. The second component is the characterization of the Nrf2-mediated stress response. Treatment with tBHQ and/or Nrf2 siRNA gave statistically significant changes in the expression of a subset of 11 proteins. Treatment with oxidized LDL gave statistically significant increases in the expression of 7 of those 11 proteins plus one additional protein. All of the oxLDL-mediated increases were attenuated by Nrf2 siRNA. These results reveal a specific, multifaceted response of the foam cells to the incoming toxic oxidized LDL.

## Introduction

The oxidative hypothesis of atherosclerosis is based on the observation that oxidized low density lipoprotein (oxLDL) is taken up by macrophages in an unregulated manner to produce lipid-laden foam cells [1], [2], [3]. The unregulated uptake of oxidized LDL occurs through a variety of scavenger receptors, many of which are found in other cell types [4], [5], [6], [7]. Non-scavenger receptor mediated uptake of LDL to form foam cells has also been reported [8]. Foam cells are the main component of the fatty streaks that are the earliest recognizable form of atherosclerotic lesions and are also found in mid- and late-stage lesions [9], [10]. Foam cells actively participate in the development of vascular disease by increasing inflammatory cytokine production, increasing the production of reactive oxygen species, stimulating vascular smooth muscle cell proliferation, and increasing lipid deposition via increased scavenger receptor expression [11], [12], [13], [14], [15]. Based on these various actions, foam cells must be considered key contributors to both the initiation and progression of atherosclerosis.

A notable paradox in the cell biology of foam cells is that oxidized LDL is toxic and contains a diverse set of toxic species, such as oxidized cholesterol, aldehydes, and lipid hydroperoxides [16], [17], [18]. In fact, one part of the initial recognition of oxidized LDL as a biologically relevant type of modified LDL was the observation of its cytotoxicity [19]. Considering the active role of foam cells in the progression of the disease, it is intriguing to consider the ability of foam cells to survive the toxic effects of oxidized LDL to be an unwanted factor in the etiology of atherosclerosis. However, the molecular events that allow macrophages to load toxic oxidized LDL and form foam cells without cell death have not been fully characterized.

Recently, investigators have shown that the transcription factor nuclear factor (erythroid-derived 2)-like 2 (Nfe2l2 or Nrf2) is pro-atherogenic [20], [21]. In those experiments, Nrf2 knockout in ApoE^−/−^ mice had significantly reduced atherosclerosis. Nrf2 is ubiquitously expressed and is generally viewed as a master regulator of the cellular response to oxidative stress. Various investigations have identified a large number of Nrf2 targets using both gene array methods and bioinformatic analyses of the antioxidant response element (ARE) to which Nrf2 binds [22], [23], [24], [25], [26]. A number of those targets are antioxidant and detoxification proteins, although other targets can be classified with a wide-ranging set of biological functions. Taken together, the list of Nrf2-targets described in these reports exhibits an intriguing variance depending on both the type of stimulus and the type of cell. The variance seen in these reports also makes it clear that it is not possible to explicitly predict the effects of Nrf2 manipulation in a given system.

The goals of the experiments described here have been to develop and use a quantitative proteomic approach to determine the stress response of macrophages to oxidized LDL loading and test the ability of Nrf2 to regulate the response. Our working hypothesis is that the ability of macrophages to survive oxLDL-loading is mediated by a Nrf2-dependent stress response. Work from a number of laboratories has been using traditional proteomic methods to study changes in protein expression associated with the transition of macrophages into foam cells [27], [28], [29]. All of these reports, including experiments from our group, have used two-dimension gel electrophoresis to find differentially expressed protein bands and identified those proteins with mass spectrometry. Although this electrophoresis-based approach has been a productive tool, most investigators agree that the use of electrophoresis to detect differentially expressed protein has several limitations. These limitations have lead proteomics researchers to explore new methods in which gel electrophoresis is not the primary source of protein quantitation information.

In response, we have used a new LC-tandem mass spectrometry-based quantitative proteomic approach to study changes in protein expression. Specifically, selected reaction monitoring (SRM, also known as MRM) in a triple quadrupole mass spectrometer was used to detect and quantify a specific set of tryptic peptides as quantitative markers for the parent proteins [30], [31], [32]. Using SRM, we have established a multiplexed assay targeting a group of 28 antioxidant proteins and stress response proteins along with a group of 6 housekeeping proteins and a non-endogenous internal standard protein. Advantages of the method are the number of proteins measured in a single assay, a wide dynamic range, the fundamentally quantitative nature of the assay (all experiments used n≥5 with standard statistical tests), and the excellent precision. These features make this assay a powerful tool to carry out sensitive and comprehensive tests of large groups of proteins, including entire biochemical pathways.

The work was focused on the Nrf2-dependent components of the antioxidant enzyme system as a key element in the ability of foam cells to survive the toxicity of oxidized LDL. Our experiments used a model of foam cells in which a cultured mouse macrophage-like cell line was treated with oxidized LDL, with and without Nrf2 siRNA knockdown. Treatments with a small molecule activator of the Nrf2-pathway, tert-butylhydroquinone (tBHQ), were used for comparison. The targeted quantitative proteomic analysis then measured the antioxidant and stress response proteins to determine any changes in expression in this group. Our results showed a specific induction of a subset of antioxidant proteins. Comparison to the tBHQ-treated cells showed that not all of the potential Nrf2-targets were affected by oxLDL. These results reveal a specific, multifaceted response of the foam cells to the incoming toxic species. This response is consistent with an enhanced ability of the foam cell to survive those toxic species, allowing them to participate in the down-stream actions to continue lesion development.

## Materials and Methods

### Cells and Culture Conditions

The J774 mouse macrophage-like cell line was purchased from American Type Culture Collection (ATCC, Tib 67). The cells were maintained in Delebeco’s modified minimal essential media (DMEM) supplemented with 10% fetal bovine serum (FBS), penicillin and streptomycin, and 10 ng/mL selenium (Sigma-Aldrich).

### LDL Oxidation

Human low density lipoprotein (LDL) was purchased from Intracel (Frederick, MD) as a 5 mg/mL solution in tris buffered saline (TBS - 150 mM NaCl, 10 mM Tris, pH 7.8) with 5 mM EDTA. The LDL was dialyzed against TBS overnight at 4°C to remove the EDTA. The oxidation reaction was initiated by dialysis against TBS with 10 µM CuSO_4_ overnight at room temperature and stopped by dialysis against TBS with 5 mM EDTA overnight at 4°C.

### Cell Treatments

The cells were plated in 60 mm dishes at 4×10^5^ cells per dish and incubated overnight before any treatments. For the oxidized LDL treatments, the oxLDL preparation was diluted to 250 µg/mL in complete media (DMEM with 10% FCS, pen/strep, and selenium) and added as a 1 mL aliquot to give a final concentration of 50 µg/mL.

The siRNAs were purchased from SigmaAldrich. For Nrf2 knockdown, a pool of 3 siRNAs was used at a final concentration of 5 nM each; (1) sense GAAUUACAGUGUCUUAAUA[dt], antisense UAUUAAGACACUGUAAUUC[dt], (2), sense GUGAAAUGCAGAAACACUU[dt], antisense AAGUGUUUCUGCAUUUCAC[dt], (3) sense GAAACCUCCAUCUACUGAA[dt], antisense UUCAGUAGAUGGAGGUUUC[dt]. Stock solutions were prepared in TE buffer (10 mM tris, 1 mM EDTA, pH 8). Catalase knockdown used an esiRNA from SigmaAldrich at a final concentration of 10 ng/mL. For the transfections, InterferIn siRNA Transfection Reagent (PolyPlus) was used. The cells were treated for 48 h with both the siRNA and esiRNA systems.

The Nrf2 stabilizing reagent tert-butylhydroquinone (tBHQ) was prepared as a stock solution in 95% ethanol. The stock solution was diluted in two steps with complete media and added as a 0.5 mL aliquot to give a final concentration of 25 µM. Treatments were for 24 h.

The changes in Nrf2 expression produced by these treatments, an increase in expression with tBHQ-treatment that is blocked by Nrf2 siRNA pre-treatment, were verified by Western blot analysis (Figure S1).

### Preparation of Protein Samples

The cells were harvested by scraping into ice cold TBS and pelleted by centrifugation at 500 g for 5 min. The cell pellet was suspended in TBS containing a protease inhibitor cocktail (Sigma-Aldrich), incubated for 10 min on ice, and repelleted by centrifugation. The cells were then suspended in 1 mL water, mixed well with a pipet, and split into two 0.5 mL aliquots. One aliquot was mixed with 0.5 mL 2% SDS in a 50 mM tris, 5 mM MgCl_2_, pH 7.6 and heated at 80°C for 15 min to completely lyse the cells for proteomics. DNAse and RNAse were added to break-up the viscosity. Protein concentrations were measured using a detergent compatible reagent kit (BioRad DC Protein Assay). The second aliquot was mixed with 0.5 mL 100 mM phosphate buffer, pH 7.5 and sonicated to completely lyse the cells for the enzyme assays.

### Short-run SDS-Page and In-gel Tryptic Digestion

Aliquots of the protein samples in SDS containing 100 µg total protein were desalted by precipitation in 1mL of acetone overnight at −20°C. The protein pellet was solubilized in 100 µL Laemmli sample buffer and 20 µg protein loaded in a 12.5% SDS-Page gel (BioRad Criterion system). The gel was run for approximately 15 min at 150 V to give a 1.5 cm gel. The gel was fixed in 50% ethanol, 40% water, 10% acetic acid for 30 min, washed with several changes of water, and stained for 5 min with Coomassie blue (GelCode blue, Pierce Chemical Company).

Each lane was cut as a single sample and the gel piece divided roughly into 8–10 pieces. The gel pieces were destained in 50% ethanol, 40% water, 10% acetic acid overnight at 50°C with several changes as needed for complete destaining. A standard in-gel digestion method was used [33]. Briefly, proteins were reduced with DTT and alkylated with iodoacetamide (15 mg/mL and 30 mg/mL in 10 mM ammonium bicarbonate, respectively) for 20 min each. The reduction and alkylation reagents were removed and digestion was carried out by adding 1 µg trypsin (Promega) in 200 µL 10 mM ammonium bicarbonate for overnight at room temperature. The peptides produced were collected by extraction in 200 µL 50% ethanol, 50% water with 1% formic acid. The extract was evaporated to dryness and reconstituted in 150 µL 1% acetic acid in water for LC-tandem MS analysis.

### Liquid Chromatography-tandem Mass Spectrometry

The LC-tandem MS system was a TSQ Vantage triple quadrupole mass spectrometry system (ThermoScientific) with a splitless nanoflow HPLC system with autoinjector (Eksigent). A 10 cm C18 column (Phenomenex Jupiter) packed in a fused silica electrospray tip (New Objective) was used. 5 µL to 10 µL Volumes of the samples were injected and loaded onto the column at 2 µL/min with 0.1% formic acid. The column was eluted at 160 nL/min with a linear gradient of CH_3_CN in water with 0.1% formic acid (3% CH_3_CN to 63% CH_3_CN in 30 min).

The triple quadrupole mass spectrometer was operated in the selected reaction monitoring (SRM) mode. Ion source conditions were: spray voltage = 2.5 kV, ion transfer tube temperature = 300°C, positive ions. Collision induced dissociation conditions were: Q1 and Q3 resolution = 0.7Da, collision cell pressure = 1mTorr, collision energy dependent on the m/z of the parent ion and optimized for each reaction, and cycle time was set for 1.0 sec. A total of 35 proteins were monitored in these experiments as shown in Tables 1 and 2. The SRM conditions were managed through the program Pinpoint (ThermoScientific) and included 2 peptides from each protein with 3 to 6 fragmentation reactions per peptide. Scheduling was used to monitor each peptide in a 4 min time window centered on the elution time of the peptide. Integrated chromatographic peak areas for each peptide were determined using the Pinpoint program. The response for each protein was calculated as the total integrated area for both peptides monitored for that protein. Data were analyzed as either this raw total integrated area and after normalization to the house-keeping proteins.

**Table 1:** Summary of proteins measured in the antioxidant protein assay.

	Gene name	Protein name	Molecular weight (kDa)	Function
1	Akr1b1	aldo-keto reductase family 1 member 3	36	aldehyde reduction
2	Aldh2	aldehyde dehydrogenase 2, mitochondrial	57	aldehyde oxidation
3	Cat	catalase	60	peroxidase
4	G6pd	glucose-6-phosphate dehydrogenase	59	source of NADPH
5	Gpx1	glutathione peroxidase 1	22	peroxidase
6	Gpx4	glutathione peroxidase 4 isoform 1	29	peroxidase
7	Gsr	glutathione reductase	53	glutathione reduction
8	Gsta3	glutathione S-transferase, alpha 3	25	detoxification
9	Gstm1	glutathione S-transferase, mu 1	26	detoxification
10	Gstp1	glutathione S-transferase, pi 1	23	detoxification
11	Hspa1a	heat shock 70 kDa protein 1B	70	response to stress
12	Hsp90b	heat shock protein 90 alpha (cytosolic)	92	response to stress
13	Hspa9	stress-70 protein, mitochondrial	69	response to stress
14	Hspa5	heat shock 70kD protein 5	72	response to stress
15	Msra	methionine sulfoxide reductase A	25	oxidized protein repair
16	Nnt	nicotinamide nucleotide transhydrogenase	114	source of NADPH
17	Phb	prohibitin	29	stress response
18	Phb2	prohibitin 2	29	stress response
19	Prdx1	peroxiredoxin 1	22	peroxidase activity
20	Prdx2	peroxiredoxin 2	22	peroxidase activity
21	Prdx3	peroxiredoxin 3	28	peroxidase activity
22	Prdx4	peroxiredoxin 4	31	peroxidase activity
23	Prdx5	peroxiredoxin 5	22	peroxidase activity
24	Prdx6	peroxiredoxin 6	24	peroxidase activity
25	Sod1	superoxide dismutase 1, soluble	16	superoxide reduction
26	Sod2	superoxide dismutase 2, mitochondrial	24	superoxide reduction
27	Txn1	thioredoxin 1	12	oxidized protein repair
28	Txnrd1	thioredoxin reductase	55	thioredoxin reduction

**Table 2:** Housekeeping proteins monitored to demonstrate equal loading of the gel and consistent responses in the LC-tandem MS analysis.

	Gene name	Protein name	Molecular weight (kDa)	Function
1	Calr	calreticulin	53	endoplasmic reticulum calcium binding protein
2	Gapdh	glyceraldehyde-3-phosphate dehydrogenase	36	cytosolic enzyme in carbohydrate metabolism
3	Hspd1	60 kDa heat shock protein, mitochondrial	58	mitochondrial chaperonin
4	Ldha	lactate dehydrogenase A	36	cytoplasmic enzyme in anaerobic glycolysis
5	Lyz	chicken lysozyme	16	non-endogenous internal standard
6	Ncl	nucleolin	76	nuclear RNA and DNA binding protein
7	Rps8	40S ribosomal protein S8	24	structural constituent of the ribosome

### Enzyme Activity Assays

The activities of catalase and lactate dehydrogenase were assayed in the aliquot of the sample made up in 50 mM phosphate buffer, pH 7.6 and sonicated. For the catalase activity assay, the consumption of hydrogen peroxide was monitored at 240 nM. The amount of hydrogen peroxide consumed was calculated using a molar extinction coefficient of 43.6 M^−1^cm^−1^. For the lactate dehydrogenase activity assay, the conversion of NADH to NAD^−^ was monitored at 340 nM using pyruvate as the substrate. The amount of NADH consumed was calculated using a molar extinction coefficient of 6300 M^−1^cm^−1^.

### Western Blot Analysis

Samples (20 µg protein) were separated in a 12.5% SDS-Page gel and transferred to a PVDF membrane. After washing and blocking, the membranes were incubated with the respective primary antibodies as follows: goat anti-Hspd1 (Santa Cruz Biotechnology) at 1∶200 for 1 h at room temperature, and a custom-made rabbit anti-Cat at 1∶200 for 1 h at room temperature. The secondary antibodies (conjugated to horseradish peroxidase, Pierce) at 1∶20000 were applied for 45 min at room temperature. The blots were developed using a chemiluminescent reagent system (Supersystem West Pico Chemiluminescent Substrate, ThermoFisher) and the images recorded with film.

## Results

### Design of the Antioxidant Protein Group Assay

A total of 28 antioxidant enzymes and stress proteins were included in this assay. An additional 6 proteins were monitored as housekeeping proteins along with chicken lysozyme as a non-endogenous internal standard. These proteins are listed in Tables 1 and 2. The primary goal of assembling this group of proteins was to measure the most common antioxidant enzymes plus any ancillary proteins needed to support their activities, such as the regeneration of NADPH. In the course of assembling this assay, a small number of additional proteins were added as general stress response proteins in an attempt to add other information to the results.

The peptide selection process sought to identify the group of detectable peptides for each protein with the highest signal intensity. The selection process tended to favor what can best be described as archetypal tryptic peptides, mostly in the range of 6- to 15-mers, which gave predominantly doubly charged molecular ions. The only amino acid automatically eliminating a peptide from consideration was methionine due to the variable oxidation.

The design process for the SRM descriptors for each protein was significantly facilitated by additional data from experiments performed on ion trap mass spectrometry systems (either an LCQ or LTQ). In nearly all cases, these experiments were standard protein identification experiments in which a given protein was identified based on the detection and characterization of a number of peptides in a data-dependent experiment. The results of these experiments were maintained in a database which was mined to find relevant datasets to design the SRM descriptors. In a small number of cases, either no identification or only a limited identification result was available. In these cases, the ion trap instrument was programmed to detect peptides predicted by an in silico digestion of the protein and the proper CID spectra located by searching the data set. Ultimately, all of the peptides selected for this antioxidant protein assay had high quality CID spectra recorded on an ion trap system using similar LC conditions. As a result, the details of the respective CID spectra were known and verifiable. More importantly, the elution pattern of all peptides in this assay was also known giving absolute confidence the SRM assay correctly detects the proper peptides.

**Figure 1:**
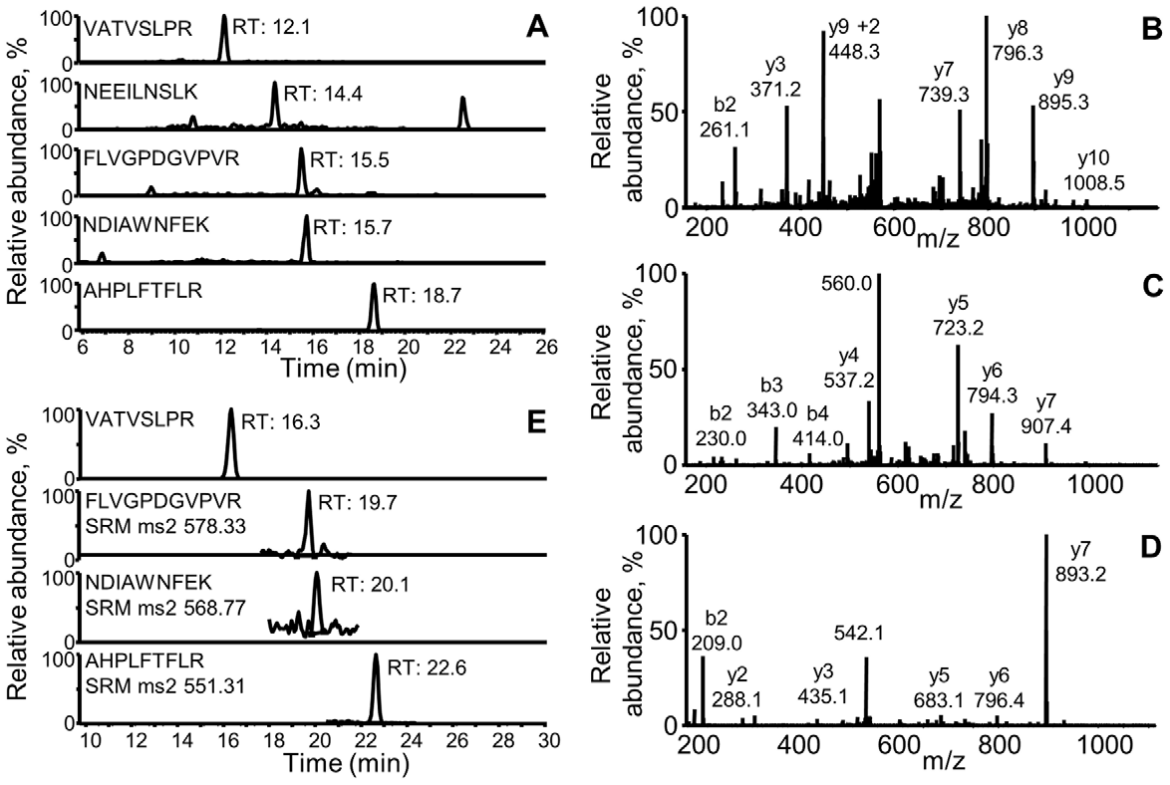
An example of the development of the SRM descriptor for a target protein. (A) The identification of glutathione peroxidase 1 (Gpx1) on a capillary column LC-ion trap mass spectrometry system (LTQ-xl, ThermoScientific) in a sample of mouse liver was evaluated to select the best peptides for inclusion in the assay. The chromatographic conditions, including column, solvent program, and flow rate, were comparable to that used on the triple quadrupole system for the final analyses. As a result, a characteristic elution pattern was obtained. (B,C, and D) From the ion trap instrument, full scan collision induced dissociated (CID) spectra were acquired which allows the identification of the respective peptides to be verified. The CID spectra for the peptides (B) FLVGPDGVPVR, (C) NDIAWNFEK, and (D) AHPLFTFLR were obtained. (E) These chromatographic and CID spectra data were used to develop the Gpx1 SRM descriptor that includes parent and product ion *m/z* to monitor, optimized collision energies for each fragmentation reaction, and a scheduled window for elution. Eventually, only two of the peptides, NDIAWNFEK and AHPLFTFLR were retained in the final assay. A similar approach was used for all other proteins in the antioxidant and stress protein group assay.

An example of the validation of an SRM descriptor, for glutathione peroxidase 1 (Gpx1), is shown in Figure 1. Based on a previous analysis in which Gpx1 was identified, only 2 potential peptides were seen. In silico analysis of the amino acid sequence of Gpx1 added two additional peptides to give a candidate set of 4 peptides. Full scan CID spectra of these peptides were acquired on the linear ion trap instrument in an experiment that targeted the doubly charged molecular ions. The results of these experiments validated both the retention pattern of the peptides (Figure 1a) and the CID spectra (Figure 1b–d). Subsequent testing on the triple quadrupole system with a variety of samples allowed the Gpx1 descriptor to be reduced to 3 peptides selected for the highest signal intensities (Figure 1e). With use, this descriptor was finally limited to the AHPLFTFLR and NDIAWNFEK peptides based on the highest signal intensity and least variability.

The complete descriptor used for this 35 protein assay is shown in supporting Table S1.

### The Use of Short-run Gel Electrophoresis to Enhance Sample Processing

Complete and reproducible protein digestion is critical for the success of these analyses. Complete digestion not only enhances the sensitivity of the assays by generating this greatest amount of peptide per protein possible but also enhances the reproducibility by taking the digestions to completion. Effective digestion of a protein requires both complete denaturation and the reduction-alkylation of any disulfide bonds. In gel digestion facilitates these processes. The SDS-Page separation uses highly denaturing conditions to assure the best possible access to protease digestion sites. Proteins immobilized in an SDS-Page gel can be efficiently reduced and alkylated prior to digestion. This step not only further enhances the protease function, but also allows the use of cysteine-containing peptides in the assay. All reagents used in the reduction and alkylation are then simply washed away, preventing any deleterious effects on the protease or in the subsequent LC-tandem MS analyses.

**Figure 2:**
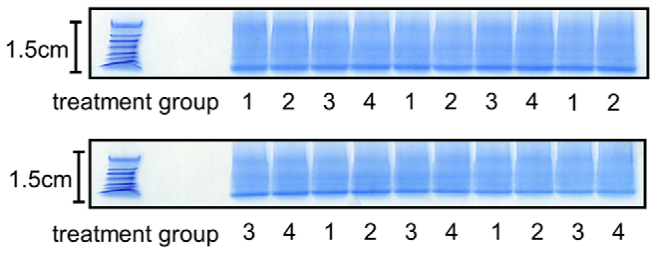
Short run gel electrophoresis used for sample preparation and in-gel digestion. A 12.5% polyacrylamide gel was used to immobilize the samples and facilitate a highly reproducible in-gel digestion. Each lane contains 20 µg total protein. The gel was run at 150 V for 15 min to give a length of approximately 1.5 cm. After fixing, the gel was stained for 5 min. The staining pattern shows equal loading of each sample. Each lane was then cut as a single sample for processing via a typical in-gel digestion protocol. The treatment groups in this gel are: 1, control; 2 −+oxLDL; 3+Nrf2 siRNA; 4+both. Each treatment was n = 5 to give a set of 20 samples making two gels necessary.

Our approach to the SDS-Page component is not the traditional separation experiment. As seen in Figure 2, the samples were run only 1.5 cm into an SDS-Page gel, fixed, and stained. The utility of the short-run gel electrophoresis is to immobilize the protein to take advantage of in-gel digestion. Minimizing the length of the gel makes it possible to keep a single digest per sample and avoid any impractical increases in the scale of the digestion. No specific quantitative information is taken from the gel appearance, although gross problems with sample quality such as large differences in sample amounts might be noticeable in the staining pattern.

### Monitoring Selected Housekeeping Proteins to Assess Equal Loading and Consistent Mass Spectrometry Responses

A component of this assay is the use housekeeping proteins to demonstrate equal loading of the samples and for signal normalization as needed. Some form of normalization is traditionally used in quantitative mass spectrometry experiments to account for factors such as variations in yield of the analyte in the sample preparation process and variations in the response of the mass spectrometer, both sample-to-sample and day-to-day. There are many different approaches to this normalization process, including the addition of stable isotope labeled-analogues and non-endogenous analogues as internal standards. The use of endogenous housekeeping proteins taken here reflects a common approach used in the quantitation of proteins in biological samples. Western blot analyses always contain the parallel analysis of some housekeeping protein to demonstrate equal loading. Similarly, quantitative rtPCR methods use the parallel analysis of a housekeeping gene or genes for normalization.

This assay incorporates a set of 6 housekeeping proteins; calreticulin (Calr), glyceraldehyde-3-phosphate dehydrogenase (Gapdh), heat shock protein 60 (Hspd1), lactate dehydrogenase (Ldha), nucleolin (Ncl), and ribosomal protein S8 (Rps8). The selection of these proteins was largely empirical and based not only on work with cultured cells but also other experiments analyzing mouse heart and liver. The key criteria for the selection of a housekeeping protein were a lack of change under the various treatment conditions and an abundance that makes them easily detectable without being far greater than the target proteins. The housekeeping proteins also represent a variety of subcellular localizations, including cytosol, mitochondria, nucleus, ribosomes, and endoplasmic reticulum, respectively, although many proteins reside in a variety of locations. In addition, practical consideration also played a role – such as access to a reliable antibody for Hspd1 and experience with an activity assay for Ldha. It is certain a given set of housekeeping proteins will not be applicable to all experiments. Therefore, validation of the housekeeping choices should be a component of any experiment.

**Figure 3:**
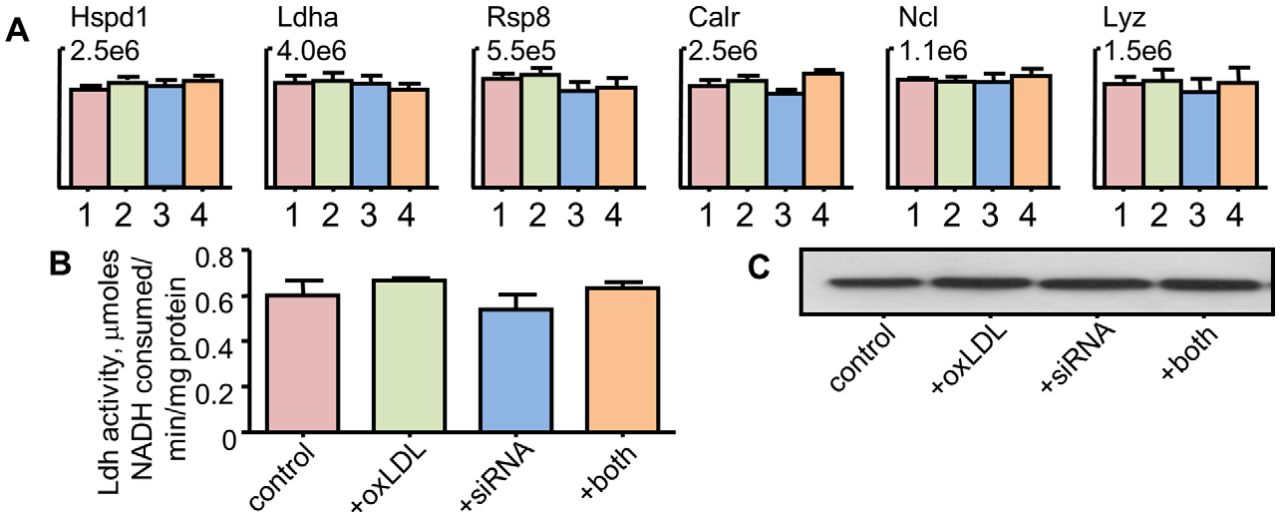
Equal content of the housekeeping proteins validated by a variety of approaches. A) Statistical analysis of the response in the selected reaction monitoring assay. Data for five of the housekeeping proteins (Hspd1, Calr, Rsp8, Ldha, and Ncl) and the internal standard (Lyz) plotted as the raw signal strength for all samples (mean ± SEM, m = 5). No statistically significant change was seen across the four treatment groups for any protein by either a Students t-test or a one-way analysis of variance. B) Enzyme activity analysis for lactate dehydrogenase in all samples. No statistically significant changes were seen across the four treatment groups by either a Students t-test or a one-way analysis of variance. C) Western blot analysis of Hspd1 showing equal content in all treatment groups. Representative samples from a control, oxidized LDL-treated (+oxLDL), Nrf2 siRNA-treated (+Nrf2 siRNA), and treated with both Nrf2 siRNA and oxidized LDL (+both).

The validation of the housekeeping proteins was based primarily on observations within the assay, with additional data from Western blot analysis and an enzyme activity assay. As shown in Figure 3a, the principal evaluation is the consistency of signals for the 5 housekeeping and internal standard protein. For all of these proteins in this set of data, a one-way analysis of variance with a multiple comparisons was used. As a group, the data in Figure 3a show the combination of equal samples loading, consistent sample preparation, and consistent mass spectrometer response. Additional analyses by Western blot for Hspd1 and an enzyme activity assay for Ldha verified the primary statistical approach. This consistency means the primary comparisons of expression for each protein can be based directly on the mass spectrometry signals.

### Ion Suppression Effects

Because of the complexity of the samples and the desire to inject as much material as possible to enhance sensitivity, the possibility of signal suppression was considered. Ion suppression occurs when competition exists between the parts of a mixture, whether among analytes or matrix components, in a way that attenuates signal for the analytes. To test for suppression effects, increasing amounts of material were injected based on a systematic dilution of a set of independent samples (n = 5). Dilutions were made so peptides from the equivalent of 0.25, 0.50, 0.75, 1.00, and 1.25 µg of total protein were injected in a 10 µL sample. The signals were recorded for all proteins detected in the sample (target proteins and housekeeping proteins).

**Figure 4:**
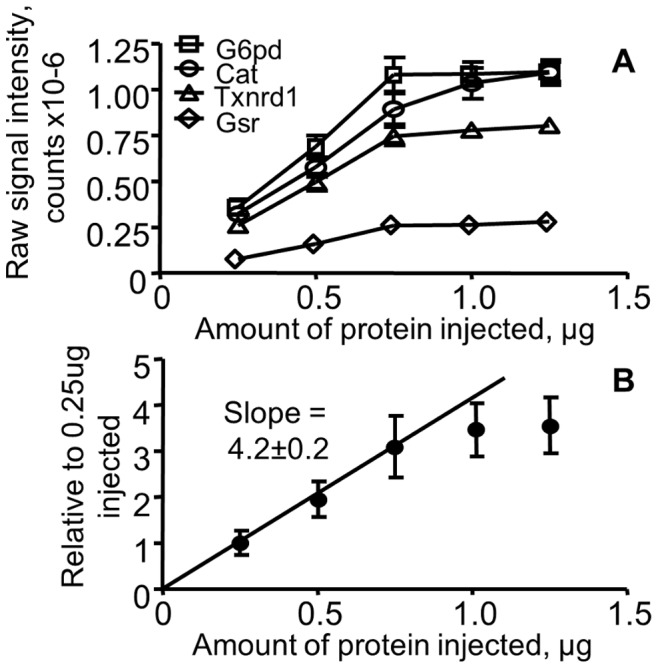
Possible ion suppression tested through a range of sample loading amounts. Increasing amounts of total protein equivalents ranging from 0.25 µg to 1.25 µg were injected. A) Responses for glucose-6-phsophate dehydrogenase (G6pd), catalase (Cat), thioredoxin reductase (Txnrd1), and glutathione reductase (GSR) plotted using the raw abundance data from the SRM experiment (mean ± SEM, n = 5). B) The average response for all proteins monitored in this experiment. For this representation, the SRM responses for each protein were normalized to the average values for the respective 0.25 µg injection. This normalization gave an expected slope of 4.0 through the linear range.

The suppression data were analyzed in two ways (Figure 4). The first analysis method simply looked at the average signal for each protein. Figure 4a shows the results for 4 representative proteins. For each, signal intensity was not linear throughout the entire range of injection amounts tested, although a significant linear range from 0.25 to 0.75 µg protein injected was observed. The second analysis normalized the signal for each protein to the average signal for the 0.25 µg injection (Figure 4b). The goal of this normalization was two-fold, it allowed all detected proteins to be considered as a set on the same scale and it allowed comparison to expected ratios of 1.0, 2.0, 3.0, 4.0, and 5.0 for the different dilutions. This analysis again shows that the signal intensity increases linearly through the 0.75 µg load but higher loads did not continue the expected increases. Importantly, linear regression of these data gives the expected slope (4.0±0.2) and extrapolates to within experimental error of 0 (x-intercept = 0, y-intercept = 0). Therefore, all experiments use injection amounts of 0.5 µg total protein equivalent.

### Precision Tests

The precision of the assay was determined at both in-run and sample-to-sample levels. The in-run precision of the assay was tested with a pooled sample analyzed multiple times (n = 8). This precision test had two components. The first component was a determination of the in-run precision of the entire assay. For the proteins covered in the assay and detected in the J774 cells, the average relative standard deviation was 7.2% with a range from 2.1% to 24.3%. These results include three proteins, Gpx4, Gsta3, and Nnt, which were near the limits of detection in these samples and had relative standard deviations>20%. Excluding these three low abundance proteins, the average relative standard deviation was 5.8% with a range from 2.1% to 10.7%.

In order to test for any need or advantage in limiting the number of target proteins covered in the assay, the in-run precision was also determined for a modified assay focused on a sub-set of only 10 proteins (Table 3). These proteins included examples of higher signals (Hspd1, Ldha, and Prdx1), intermediate signals (Akr1b1, Cat, Sod1, and Txn1), and lower signals (G6pd, Sod2, and Txnrd1). When the analysis was limited to just these 10 proteins, the number of transitions monitored was reduced from 317 to 98. Because the cycle time was still specified as 1 sec, this reduction increased the dwell time by approximately 3-fold. Average precision for these proteins in the limited analysis was 3.3% with a range of 1.5% to 5.3%. Therefore, including the larger number of proteins did lead to a statistically significant, albeit quantitatively modest, increase in the relative standard deviation of the assay.

**Table 3:** In-run precision.

	Full assay monitoring 317 fragmentation reactions covering 35 proteins	truncated assay monitoring 98 fragmentation reactions covering 10 proteins
Gene name	Relative standard deviation	Relative standard deviation
Prdx1	5.6%	3.1%
Ldha	3.6%	2.0%
Txn1	4.7%	3.1%
Hspd1	5.5%	4.4%
Cat	6.4%	1.5%
Akr1b1	9.1%	4.7%
G6pd	7.0%	5.3%
Sod1	10.3%	2.8%
Txnrd1	8.2%	3.5%
Sod2	7.8%	2.8%
	average for 10 selected proteins = 6.8%average for all 36 detectableproteins = 7.2%	average for 10 selected proteins = 3.3%

The sample-to-sample precision was tested by preparing and analyzing a set of 5 replicates of a single cell homogenate. These replicate samples were taken through the entire analytical procedure, including the short-run SDS-Page, in-gel digestion, and analysis. For the entire set of proteins, the average relative standard deviation was 9.4%, with a range of 2.4% to 23.8%. As with the run-to-run precision, the proteins with highest sample-to-sample variation were low abundance proteins near the limit of detection of the assay.

### Demonstration of Accuracy

**Figure 5:**
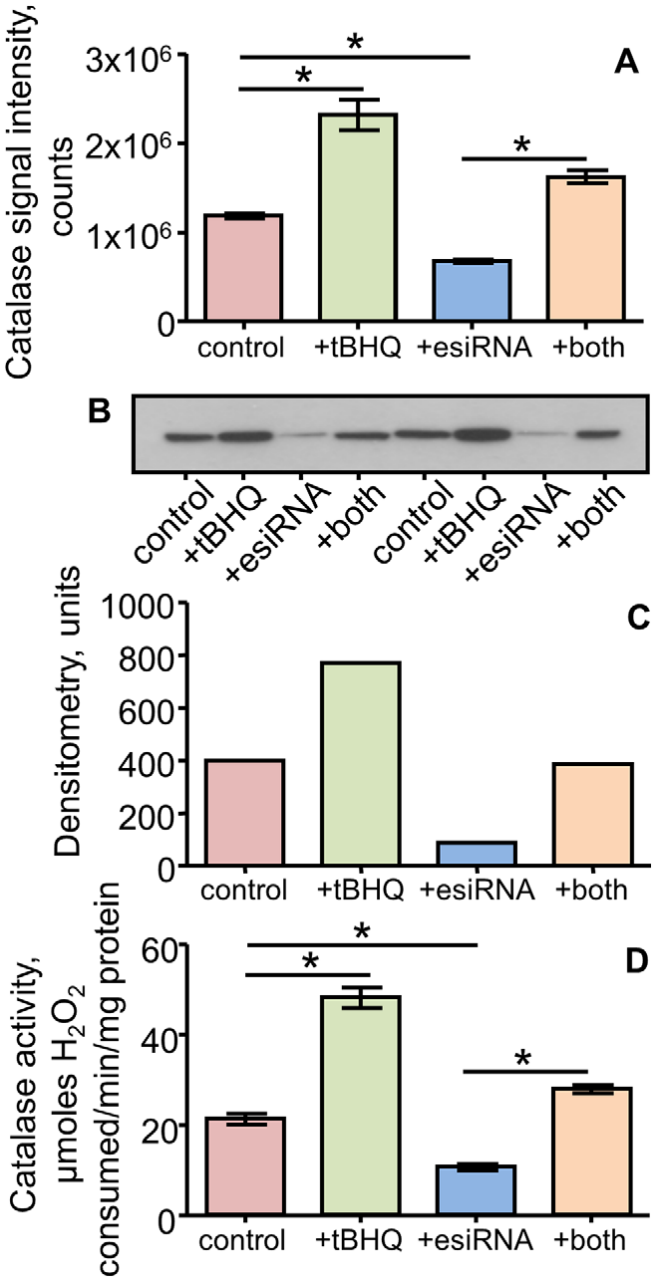
A demonstration of accuracy for the selected reaction monitoring result for catalase. The changing expression of catalase (Cat) in J774 cells treated with tBHQ, Cat esiRNA, or a combination of both was tested by several methods. A) A summary of the SRM results. The SRM signal intensities were determined for all samples in each group and are plotted as the mean ± SEM, n = 5. B) Analysis of the catalase expression by Western blot analysis with densitometry shown in (C). Two representative samples from each group were selected for analysis. The densitometry values are plotted as the average for the two determinations. D) Catalase enzyme activity assay. For A) and D) ANOVA with a Tukey multiple comparisons test was used and statistically significant differences are noted (p<0.05, n = 5).

A final component of the development experiments was to demonstrate the accuracy of the analysis. The approach taken was to use a combination of stimulation and knockdown to show that the SRM results agreed with orthogonal measurements, in this case enzyme activity assays and Western blot analysis. The target of the experiments was catalase, one of the Nrf2-targets identified below. The stimulation experiments used tBHQ and the knockdown used esiRNA with the results shown in Figure 5. Treatment of the J774 cells with tBHQ produced a statistically significant increase in catalase expression as determined by SRM analysis (Figure 5A). Treatment with catalase esiRNA not only gave a significant decrease in catalase expression but also blocked the tBHQ-dependent stimulation. Western blot analysis, used for a subset of the samples, showed a similar pattern (Figure 5B and C), both in the raw blot images and with analysis using densitometry. Finally, the analysis of the catalase activity is shown in Figure 5D. The activity assay, like the Western blot, corroborated the SRM results with increased activity following tBHQ treatment, reduced activity with esiRNA treatments, and a significant reduction of the tBHQ effect in cells pretreated with the esiRNA.

### Identification of the Nrf2-dependent Changes in Antioxidant Protein Expression Induced by Oxidized LDL

**Figure 6:**
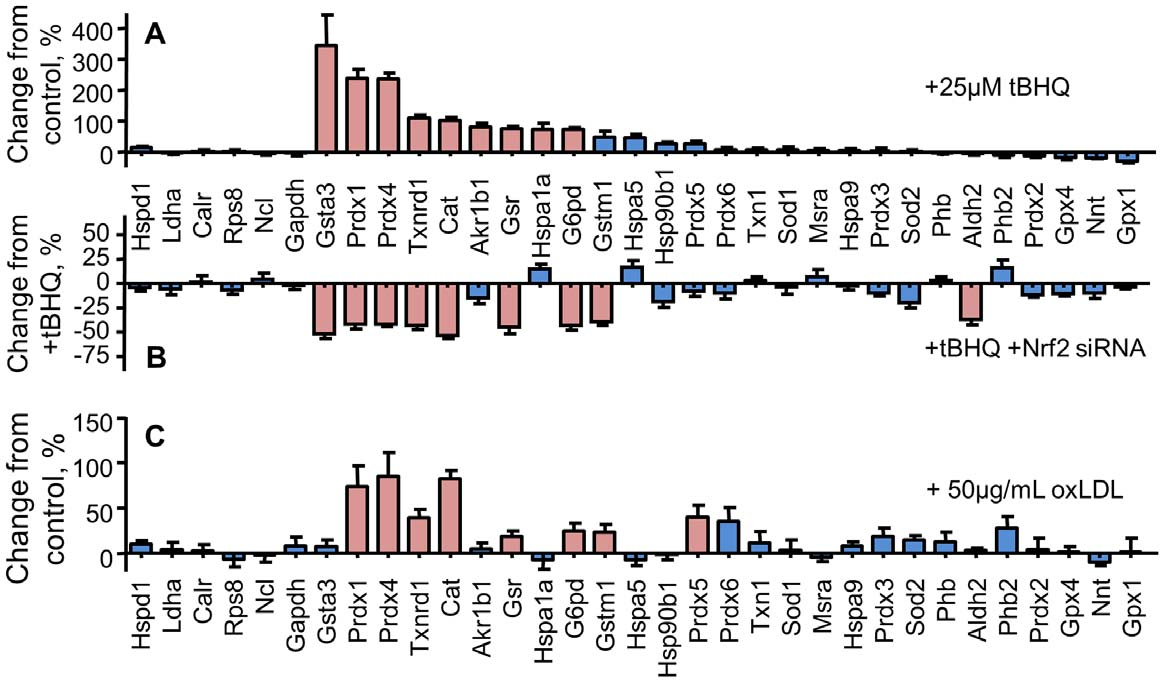
Identification of proteins induced in J774 cells treated with tert-butyl hydroquinone (tBHQ) and oxidized LDL (oxLDL). A total of 28 proteins were monitored in the assay and data are presented for the proteins that were detected. These data utilize the raw SRM signal intensity for each protein in the LC-tandem MS analyses. The SRM intensity values for the treated samples were then normalized to respective comparison group. The corresponding percent change relative to the control group is shown. A) Treatment with tBHQ (25 µM for 24h) was used to identify a subset of Nrf2-target proteins. After the six housekeeping proteins, the proteins targeted in the assay are presented in an order determined by the magnitude of the tBHQ effect. B) Treatment with Nrf2 siRNA (15 nmol/mL for 48 h) followed by tBHQ (25 µM for 24 h) was then used to verify the Nrf2-dependence. C) Treatment with oxLDL (50 µg/mL for 24 h) was compared to this signature. For each treatment, the proteins with statistically significant changes in expression are shown as a red bar (mean ± SEM, n = 5, p<0.05, Student’s t-test). Proteins with unchanged expression are shown as a blue bar.

The first series of experiments used combinations of tBHQ- and Nrf2 siRNA-treatment. The tBHQ stabilizes Nrf2 by disrupting its interaction with Keap1 in a manner that prevents proteasome degradation and enhances its translocation to the nucleus. The goal of the tBHQ-treatment was to generate an expected set of Nrf2-dependent changes by using a reagent commonly found in other studies. Treatment with 25 µM tBHQ for 24 h showed statistically significant upregulation of 9 proteins measured in this assay, with expression increases ranging from approximately 60% to over 300% (Figure 6A). No statistically significant reductions from the tBHQ treatment were observed. It was interesting to see the variable effects of the Nrf2 siRNA pretreatment (Figure 6B). Knockdown of Nrf2 with the siRNA gave statistically significant reductions in the tBHQ-driven increases in the majority of cases, but the overlap with the tBHQ increase was not complete. For example, Akr1b1 and Hspa1a were increased by the tBHQ treatment but Nrf2 knockdown did not affect this increase. In addition, Gstm1 and Aldh2 were not increased by tBHQ treatment but the Nrf2 siRNA pretreatment gave statistically significant decreases in their expression. These differences illustrate the difficulties of fully elucidating the Nrf2 response, but taken together they give a signature of potential Nrf2-targets in the J774 cells.

**Figure 7:**
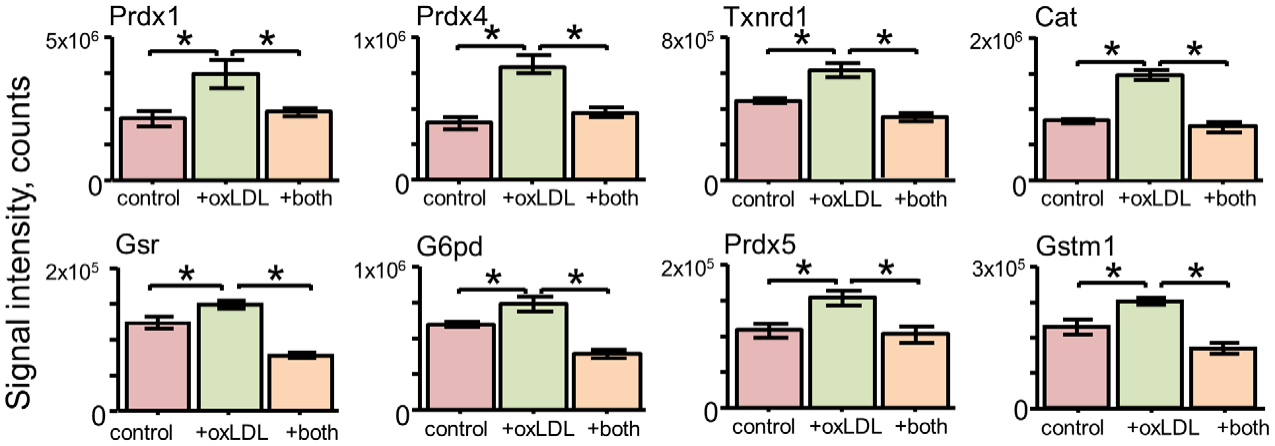
Combined effects of oxLDL- and Nrf2 siRNA-treatment on protein expression. Data are shown for effects of Nrf2 siRNA pretreatment on the 8 proteins identified as upregulated by oxLDL. For each protein, results of the SRM assay are shown for control, +oxLDL (50 µg/mL, 24 h), +both. The +both group was pretreated with Nrf2 siRNA (15 nmol/mL) for 48 h prior to the oxLDL treatement. Statistically significant differences are designated with an * (p<0.05, n = 5, ANOVA with a Tukey multiple comparisons test).

The effects of oxLDL on the cultured macrophages was then determined in light of this potential Nrf2-dependent signature (Figure 6C). Treatment of the macrophages with 50 µg/mL oxLDL for 24 h gave statistically significant increases in 8 proteins, with the magnitude of the increases ranging from approximately 25% to nearly 100%. The majority of these proteins were identified in the tBHQ- and tBHQ+Nrf2 siRNA-treatments (Figure 6A and 6B). In addition, Gstm1 and Prdx5 were upregulated by oxLDL but not by tBHQ, whereas Gsta3, Akr1b1, and Hspa1a were upregulated by tBHQ but not oxLDL. In this experiment, a combination of both Nrf2 siRNA-pretreatment and oxLDL-treatment was also tested (Figure 7). For all 8 upregulated proteins, pre-treatment with Nrf2 siRNA completely abolished the oxLDL effect. These results verify the role of Nrf2 in the oxLDL-mediated increases.

## Discussion

There were two primary goals for these experiments. The first was an analytical goal to develop and validate a multiplexed quantitative proteomics method focused on a broad set of antioxidant- and stress-related proteins. Such an assay would allow a wide-ranging yet direct characterization of how cells and tissues respond to oxidative stresses. The second was a biological goal to determine the role of the transcription factor Nrf2 in regulating the response of these proteins to oxLDL in macrophages. These results would provide needed insight into the antioxidant/stress response that protects macrophages from the toxic components of oxLDL and allows them to remain a key component of the atherosclerotic lesion.

### Multiplexed SRM Analysis is a Powerful Quantitative Proteomic Method

As an analytical method, the SRM experiment is a highly effective tool. The method is accurate, precise, and fundamentally quantitative. The general concept for the quantitative analysis of the proteins is relatively straight-forward, with three basic premises. (1) The in-gel digestion of a given protein with trypsin produces a reproducible set of observable peptides. (2) Those peptides can then be specifically detected in a complex mixture using the SRM approach. (3) The more abundant a protein is in the sample, the more abundant the peptides will be and the stronger the corresponding signal in the SRM experiment.

The design of the SRM conditions for each protein (referred to here as an SRM descriptor) was based on selecting a series of the most detectable peptides for a give protein. Methionine-containing peptides and peptides with a missed tryptic cleavage were excluded. Preference was given to peptides that gave doubly charged peptide ions without a significant triply charged ion, eluted in an acceptable time window, were not affected an internal KP or RP sequences, and did not contain a cysteine. Of the approximately 100 peptides monitored in this assay, the only exception to these preferences was a small number of peptides with cysteine residues. These exceptions were deemed necessary to give a sufficient number of peptides for the proteins Gpx1 and Gpx4. It is important to note that although cysteine containing peptides are not ideal because of the possible variability of the alkylation reaction, no adverse effects of the inclusion of these peptides were noted, consistent with the reduction-alkylation reactions having good reproducibility. In the development process, most SRM descriptors began with 4 or 5 peptides, with the 3 peptides giving the most intense and reproducible signals retained a second version of the descriptor before a final choice of the 2 peptides per protein with the least variability in practice. The rationale here was to find an optimal balance between a highly multiplexed assay while maintaining good dwell times in the monitoring of each reaction. We chose not to reduce the descriptors to 1 peptide per protein, believing the additional information will make the assays more robust. The only exception is Prdx4, a small protein (233 amino acids) with significant sequence overlap with Prdx1 - only 1 suitable peptide could be identified for its SRM descriptor.

All of the descriptors used in these experiments were designed based on data generated in our laboratory. This process can, however, be expedited by databases accessed by the internet. For example, the PeptideAtlas is a searchable database of human, mouse, and yeast proteins that may be used to see peptides detected for each protein and the CID spectra of those peptides [34], [35]. Although a valuable starting point, our experience strongly favors direct observation of the peptides, the resulting CID spectrum, and especially the chromatographic elution pattern for a reliable assay. In the case of some low abundance proteins, this direct observation may require access to different types of samples via some form of tissue or cell library or even the generation or purchase of standard samples through resources like commercially available transfection-ready cDNA clones and transiently transfected cell lysates.

The approach to data analysis in these experiments focused on the raw signal intensities as the most straight-forward approach possible. The use of this approach began with a traditional step that is common to a variety of measures in biological systems – the demonstration of no change in a series of housekeeping proteins. One might question how such a simple approach to the data analysis could work so well. The simplest answer is the highly controlled nature of the samples that are well-normalized and cleaned-up by SDS-PAGE to give a consistency that allows the direct recognition of differentially expressed proteins. In most experiments, however, it is likely for some type of normalization to be preferred. This normalization could use any of the housekeeping proteins or the exogenous internal standard. In these experiments, the data were also evaluated with normalization but no significant differences were seen in either the extent of changes, the precision of the results, or the statistics of the interpretation.

Recently, Aebersold’s group has proposed the estimation of absolute amounts of the different proteins in a sample based directly on the mass spectrometry results [32]. Their term for this approach is the ‘best flyer’ concept. This best flyer approach to estimating absolute amounts of proteins in a sample is based on the general tendency for the best peptides detected from a given protein to have a consistent set of traits that leads to a uniform response in the mass spectrometer. This uniformity, in turn, forms a basis for a practical estimation of protein amounts without complex internal standard sets (such as isotopically labeled versions of all peptides detected) or calibration approaches. In our experiments, we have added a non-endogenous internal standard, chicken egg lysozyme, to facilitate this process. Therefore, all mass spectrometric signals could be converted to amounts of protein based on the ratio to the lysozyme signal and the amount of lysozyme added to the sample.

Although we have validated the changes in catalase expression seen in these experiments with Western blot analysis and enzyme activity assays, it is reasonable to question whether large scale validation of mass spectrometry results by techniques such as Western blot is needed or fitting. Even modest experience with Western blot exposes researchers to the vagaries and limitations of the technique (along with its advantages). Specifically, antibodies that do not work for the protein they are designed to recognize, the observation of non-specific bands at other positions in the gel, failed or partially-failed blots, a limited dynamic range, over- and under-exposure of film, etc. The foremost issues, however, are a) most Western blot experiments have not been validated in their own right, making correlation to other techniques such as mass spectrometry of questionable value, and b) the semi-quantitative nature of Western blot means most experiments use only representative samples without replicates and statistical analyses demanded for all other types of quantitative results. These observations are not to say Western blot is a bad or flawed technique, but rather to question any requirement that it be used as the validation technique by which the mass spectrometry results are judged. As seen in these experiments, the SRM analyses are quantitative and precise. A relatively large number of biological replicates were used in all experiments and appropriate statistical methods were used to evaluate the results. Finally, one should also recognize the distinguished history of SRM analysis in quantitative assays for a broad selection of molecules. As a result, the successful use of SRM in the analysis of proteins can be seen as an evolution of a highly tested approach to a new type of analyte.

### The oxLDL-driven Antioxidant Response in Macrophages is Mediated by Nrf2

Experiments with the Nrf2-stabilizer tBHQ and Nrf2 siRNA established a group of Nrf2-targets in the macrophages. A total of 11 different proteins, covered in the assay, were either significantly up-regulated with tBHQ treatment or significantly down-regulated by Nrf2 siRNA. Of these proteins, Gsta3, Prdx1, Txnrd1, Cat, G6pd, and Gstm1 have demonstrated Nrf2 binding in a ChIP-seq experiment [36]. In addition, Akr1b1, Aldh2, Gsr, and Hspa1a have all been reported as differentially expressed based on microarray analysis in experiments designed to activate Nrf2-targets [22], [23], [24], [25]. The overlap between oxLDL treatment and tBHQ treatment was not complete. For example, oxLDL treatment gave an increase in Prdx5 expression that was attenuated by Nrf2 siRNA but Prdx5 did not responde to tBHQ treatment or the Nrf2 siRNA pretreatment. No increase was seen with oxLDL treatment in Gsta3, which is a prototypical Nrf2-responsive gene. Other proteins not altered by tBHQ or oxLDL have also been reported as Nrf2-responsive in previous experiments. These proteins include Sod1 and Txn1 [37]. Txn1, in particular, was identified in a Nrf2 ChIP-seq experiment and has an ARE site [36], [37] but is not increased in the macrophages by either tBHQ- or oxLDL-treatment. This variable dependence on either cell-type or treatment is supported by the large number of different Nrf2-targets seen in the literature. Those experiments analyzed expression at the message level, leaving the question of how these changes translate to the protein level unclear. Further, although described as a master regulator of the antioxidant response it is clear that many components of the antioxidant system are not regulated by Nrf2 and numerous non-antioxidant proteins are regulated. This exceptional variability in the Nrf2-dependent response reinforces the need to be able to directly access the response in a cell type- and treatment-dependent manner and the power of a highly multiplex SRM method to make these determinations.

Recent papers with knockout mice have shown that Nrf2 knockout protects mice against atherosclerosis [20], [21]. In both papers, the results were described as surprising based on the expectation of increased oxidative stress in the Nrf2 knockout animals leading to increased LDL oxidation and uptake into the vessel wall. These investigators both cite effects of Nrf2 on lipid uptake via CD36 [20], [21] and data are presented arguing against an important role for any antioxidant response [21]. Those data, however, came from tests in the liver and not in the macrophages. Histological analyses of the lesions noted a decrease in macrophage staining in the vessels of the knockout mice. Although certainly explainable by reduced lipid uptake decreasing foam cell formation, an alternative mechanism could be increased in foam cell death in the Nrf2-deficient macrophages when the toxic oxLDL is internalized. This potential for enhanced cell death is consistent with our data showing an attenuated antioxidant protein response with Nrf2 siRNA treatment.

In conclusion, targeted quantitative proteomic analyses were found to be an effective tool for the characterization of the changes in expression of a set of antioxidant and stress response proteins in macrophages. These analyses found a Nrf2-mediated response in macrophages to oxLDL with significant upregulation of a group of antioxidant proteins. As a group, these enzymes catalyze the breakdown of hydrogen peroxide and lipid hydroperoxides contained in oxidized LDL, the detoxification of reactive species such as aldehydes, and the regeneration of key co-factors required for these reactions. Future experiments can now begin to investigate how the inability of macrophages from Nrf2 knockout animals to respond appropriately to oxLDL might potentiate the anti-atherosclerotic effects of Nrf2 knockout on lipid uptake.

## Supporting Information

Figure S1 **The increase in Nrf2 expression with tBHQ treatment is blocked by siRNA pretreatment.** Western blot analysis was used to monitor Nrf2 expression with the different treatment conditions. The cultured J774 macrophage-like cell line was treated with 25 µM tert-butyl hydroquinone (tBHQ) for 5 h, with or without 48 h pretreatment with 15 nmol/mL Nrf2 siRNA. A) The tBHQ treatment produces a significant increase in Nrf2 expression that is blocked by the Nrf2 siRNA pretreatment. B) The housekeeping protein Hspd1 was used as a loading control. Based on densitometry, the relative Nrf2 expression in these samples was: control  =  1.0, +tBHQ = 4.6, +Nrf2 siRNA +Tbhq  =  1.8.
(TIF)

Table S1 **Full SRM descriptor used for the antioxidant and stress protein assay.** The assay includes a set of 35 proteins. For each protein the table shows the parent m/z, product m/z, collision energy, start time, end time, polarity, and peptide sequence. The ThermoScientific TSQ Vantage triple quadrupole mass spectrometry system uses this table in a comma delimited form to program the instrument for the assay. The start time and end time refer to the scheduling of the retention window in which the peptide is detected. The additional tables for each protein in this document are used to manage the retention window scheduling to take into account any changes in the elution time for a peptide and/or changes one might want to make in the width of the window. The proteins included in the assay are listed in Tables 1 and 2. Two trypsin autolysis peptides are also included in the descriptor as retention markers.
(XLSX)
