# Supplementary figures and images for: A Quantitative Proteomic Profile of the Nrf2-Mediated Antioxidant Response of Macrophages to Oxidized LDL Determined by Multiplexed Selected Reaction Monitoring
Source: PLoS One. 2012 Nov 16;7(11):e50016. doi: 10.1371/journal.pone.0050016 (PMC3500347; doi:10.1371/journal.pone.0050016)

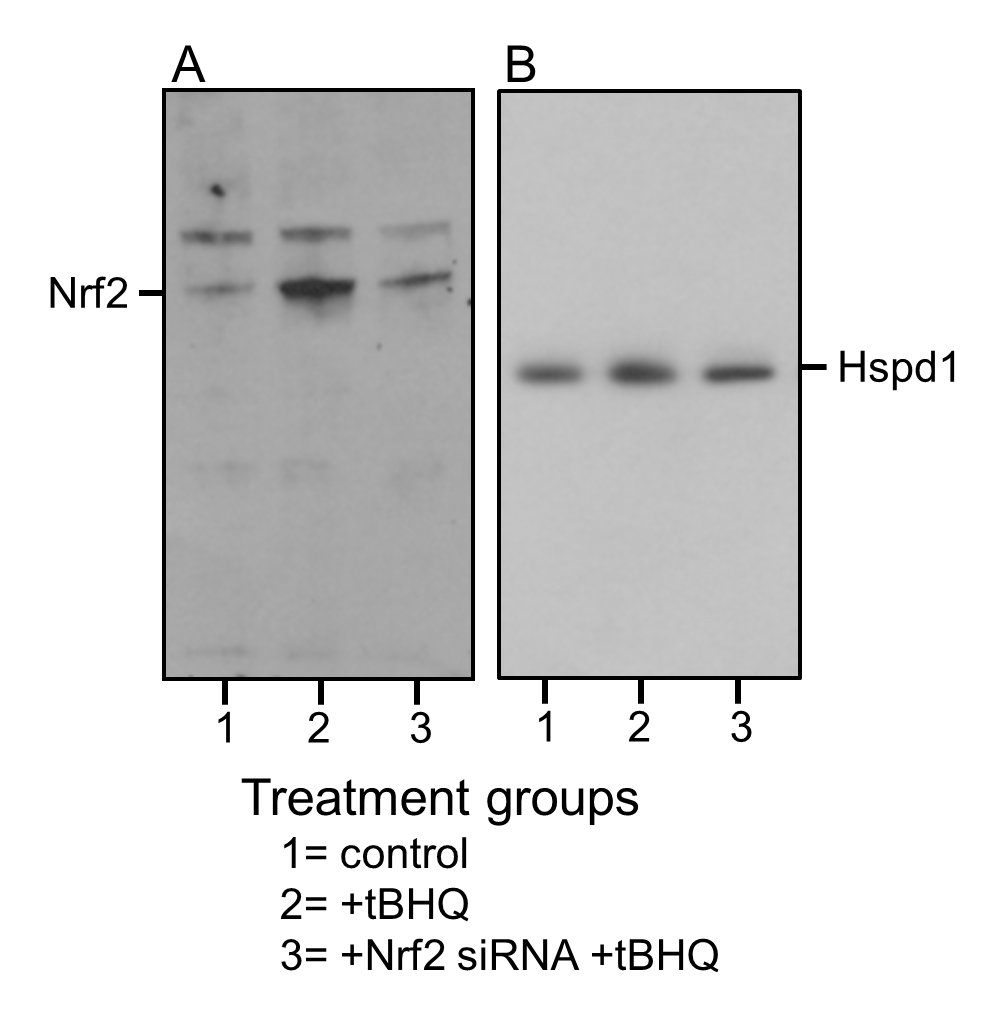

Supplement: Figure S1 — The increase in Nrf2 expression with tBHQ treatment is blocked by siRNA pretreatment. Western blot analysis was used to monitor Nrf2 expression with the different treatment conditions. The cultured J774 macrophage-like cell line was treated with 25 µM tert-butyl hydroquinone (tBHQ) for 5 h, with or without 48 h pretreatment with 15 nmol/mL Nrf2 siRNA. A) The tBHQ treatment produces a significant increase in Nrf2 expression that is blocked by the Nrf2 siRNA pretreatment. B) The housekeeping protein Hspd1 was used as a loading control. Based on densitometry, the relative Nrf2 expression in these samples was: control = 1.0, +tBHQ = 4.6, +Nrf2 siRNA +Tbhq = 1.8. (TIF) [file pone.0050016.s001.tif]
